# Drug-Coated Balloons Versus Drug-Eluting Stents for Large Vessel Coronary Artery Disease: A Meta-Analysis

**DOI:** 10.3390/jcdd12090359

**Published:** 2025-09-17

**Authors:** Jinghan Zeng, Yilu Liu, Tianlang Zhao, Jiansong Yuan, Weixian Yang, Man Wang

**Affiliations:** Fuwai Hospital, Chinese Academy of Medical Sciences & Peking Union Medical College, Beijing 100037, China; s2023002036@pumc.edu.cn (J.Z.); liuyilu616@aliyun.com (Y.L.); zhaotianlang1126@163.com (T.Z.); jsyuantg@163.com (J.Y.)

**Keywords:** drug-coated balloon, drug-eluting stent, DES, meta-analysis, large vessel, coronary artery disease, DCB

## Abstract

Objective: We aimed to conduct a meta-analysis of treatments for large vessel coronary artery disease between drug-coated balloons and drug-eluting stents. Method: We searched databases including PubMed, Web of Science, Cochrane, CNKI, and Wanfang, and selected randomized controlled trials (RCTs) or cohort studies which compared drug-coated balloons (DCBs) with drug-eluting stents (DESs). The reference vessel diameter (RVD) should be greater than 2.75 mm. The results of 12 studies with a total of 2634 patients were included in this meta-analysis. Results: The results showed that the DCB group was not inferior to the DES group in terms of the incidence of target lesion revascularization (TLR). (RR = 1.25, 95% CI: [0.84, 1.85], *p* = 0.27, I^2^ = 0%), and the incidence of bleeding events in the DCB group was lower than that in the DES group (RR = 0.30, 95% CI: [0.15, 0.59], *p* = 0.0004). The results also showed that the post-intervention minimal lumen diameter (MLD) in the DCB group was smaller than that in the DES group. (RR = −0.37, 95% CI: [−0.59, −0.16], *p* = 0.0007), but the follow-up MLD in the DCB group was not less than that in the DES group (RR = −0.03, 95% CI: [−0.14,−0.08], *p* = 0.61). Additionally the DCB group had less late lumen loss (LLL) compared with the DES group. (RR = −0.31, 95% CI: [−0.60, −0.02], *p* < 0.0001). Conclusion: This meta-analysis confirms that in the early and late stages after percutaneous coronary intervention (PCI), DCB is not inferior in efficacy and safety to DES for de novo large coronary lesions with RVD > 2.75 mm.

## 1. Introduction

Coronary heart disease (CHD) is a major cardiovascular condition affecting populations globally and a leading cause of death in both developed and developing countries. Atherosclerosis-induced coronary artery stenosis and/or thrombosis are the primary cause of angina pectoris and acute myocardial infarction [1]. Percutaneous coronary intervention (PCI) is currently the main treatment for CHD, restoring blood flow through narrowed or occluded coronary arteries and thereby minimizing damage to the heart muscle [2].

Plain old balloon angioplasty (POBA) was initially used to treat CHD but was associated with high restenosis rates due to inadequate vessel wall support at the treatment site [3]. Subsequently, coronary stents were widely adopted to reduce the risks of acute coronary occlusion and secondary restenosis following balloon dilation. Compared with bare-metal stents, paclitaxel- and sirolimus-eluting stents have significantly reduced the incidence of in-stent restenosis (ISR) [4]. Consequently, drug-eluting stent (DES) implantation has become a primary therapeutic approach for CHD. However, DES implantation can itself trigger ISR through mechanisms such as vascular endothelial injury, leading to an annual increase in target lesion revascularization (TLR) rates of 1–2% [5,6].

The drug-coated balloon (DCB) utilizes a conventional angioplasty balloon coated with anti-proliferative drugs. When positioned at the coronary stenosis and inflated, the DCB releases these drugs into the vessel wall, inhibiting intimal hyperplasia and reducing the incidence of coronary restenosis. Following drug delivery, the DCB is withdrawn, eliminating permanent vessel implantation and further minimizing restenosis risk by avoiding ongoing vascular stimulation [7]. DCB first demonstrated efficacy in treating in-stent restenosis (ISR). Bruno S, Speck U et al. established through animal studies that DCB safely and effectively inhibits intimal hyperplasia [8,9]. Subsequent clinical trials, including PACCOCATH ISR I and II, confirmed the safety and efficacy of DCB for ISR [10]. The 2023 AGENT IDE single-blind trial in the United States further demonstrated that DCB treatment for ISR resulted in lower target lesion revascularization (TLR) rates compared with conventional balloon angioplasty [11]. Consequently, DCB has emerged as the standard of care for coronary stent ISR.

As a result, the medical community has embraced the “leave nothing behind” concept and explored DCB for treating de novo coronary lesions. Byrne summarized clinical trials of DCB for de novo coronary artery disease, proposing that a DCB-only strategy may represent the future of PCI—particularly for small vessel disease, where stent-induced intimal hyperplasia is more pronounced [12]. Subsequent studies, including BELLO and BASKET-SMALL 2, further confirmed the efficacy and safety of DCB in de novo small coronary vessel lesions. Notably, the BELLO trial demonstrated significantly reduced late lumen loss (LLL) in the DCB group compared with the DES group [13,14].

Currently, robust clinical trials and comprehensive analyses on DCB use in de novo large coronary lesions remain limited. Existing studies suffer from small sample sizes, and treatment guidelines and consensus documents offer insufficient specific recommendations for this application. To address this evidence gap, this study aims to conduct a systematic review and meta-analysis evaluating the efficacy and safety of DCB for large vessel coronary artery disease. Consistent with the *Expert Consensus on Clinical Application of Drug-Coated Balloon (Second Edition)*, large coronary vessels are defined in this analysis as those with a reference vessel diameter (RVD) > 2.75 mm [7].

## 2. Materials and Methods

### 2.1. Outcome Measurements

The primary outcome is target lesion revascularization (TLR). The secondary outcomes are divided into clinical outcomes and coronary angiography outcomes. The former includes myocardial infarction (MI), cardiac death, all-cause death, target vessel revascularization (TVR), bleeding events, and restenosis, while the latter includes post-intervention minimal lumen diameter (MLD), follow-up MLD, and late lumen loss (LLL). LLL is defined as post-intervention MLD minus follow-up MLD.

### 2.2. Search Strategy

We searched the PubMed, Web of Science, Cochrane, CNKI, and Wanfang databases, and searched for keywords such as “DCB” or “drug coated balloon” or “drug coated balloon” or “drug coated balloon” and “large vessels” or “large vessels”. All databases have been retrieved up to July 2025.

### 2.3. Inclusion and Exclusion Criteria

Studies that met the following criteria were included: (1) randomized controlled trials or cohort studies; (2) the included population were patients with coronary heart disease who clinically presented with chronic coronary syndrome and acute coronary syndrome (including non-ST segment elevation myocardial infarction (NSTEMI) and ST segment elevation myocardial infarction (STEMI)), and undergoing PCI therapy; (3) comparison of DCB and DES; 4) reference vessel diameter (RVD) greater than 2.75 mm.

The exclusion criteria were as follows: (1) study with no control group or incomplete outcome data; (2) the types of lesions were peripheral vascular lesions, coronary artery bifurcation lesions, ISR, and small vessel lesions; (3) using DCB and DES in combination to treat coronary artery lesions; (4) research that cannot obtain RVD data.

## 3. Results

### 3.1. Included Studies

We found the following 12 RCTs or cohort studies that met the inclusion and exclusion criteria. The different definitions about MACE in each study are listed in Table 1.

### 3.2. Quality Assessment

Given the inclusion of numerous cohort studies, the quality of the selected literature was assessed using the Newcastle–Ottawa Scale (NOS). The NOS contains three aspects: the selection of study subjects, intergroup comparability, and the measurement of outcome indexes. The maximum achievable score is 9 points, with higher scores denoting higher methodological quality. The risk of bias assessment for each included study is presented in Table 2.

### 3.3. Primary Outcomes

**Figure 1:**
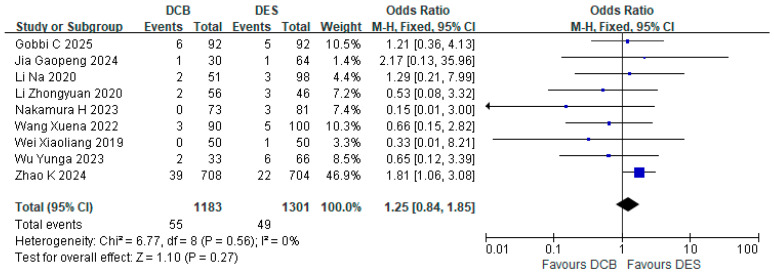
Forest plot of TLR. The squares represent the odds ratio of each individual study, with the horizontal lines indicating the 95% confidence intervals. The diamond represents the overall pooled odds ratio [15,16,18,21,22,23,24,25,26].

Nine studies reported the TLR rate. There was a total of 55 TLR events in the DCB group (*n* = 1183) and 49 TLR events in the DES group (*n* = 1301). The results showed that the TLR rate in the DCB group was not higher than that in the DES group (RR = 1.25, 95% CI: [0.84, 1.85], *p* = 0.27, I^2^ = 0%) (Figure 1).

### 3.4. Clinical Secondary Outcomes

#### 3.4.1. Myocardial Infarction (MI)

**Figure 2:**
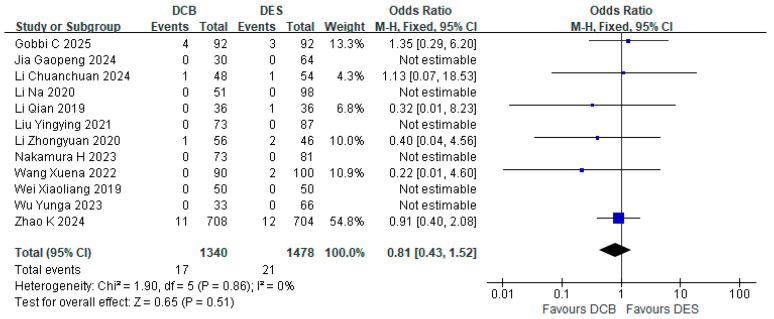
Forest plot of MI [17,18,19,20,21,22,23,24,25,26,27,28].

All 12 studies reported the incidence of MI, and in 6 studies there were no MI events in both DCB and DES groups. The remaining six studies reported 17 MI events in the DCB group (*n* = 1340) and 21 MI events in the DES group (*n* = 1478). The results showed that the incidence of MI in the DCB group was not inferior to that in the DES group (RR = 0.81, 95% CI: [0.43, 1.52], *p* = 0.51, I^2^ = 0%) (Figure 2).

#### 3.4.2. Cardiac Death

**Figure 3:**
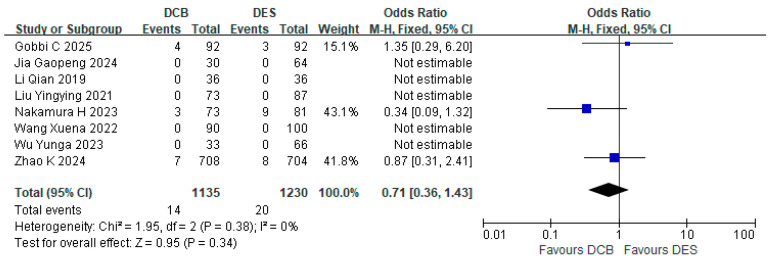
Forest plot of cardiac death [15,16,19,20,22,23,25,26].

Eight studies reported the incidence of cardiac death. The DCB group (*n* = 1135) experienced 14 cardiac deaths, while the DES group (*n* = 1230) had 20 cardiac deaths. The incidence of cardiac death in the DCB group did not significantly exceed that in the DES group (RR = 0.71, 95% CI: [0.36, 1.43]; *p* = 0.38, I^2^ = 0%) (Figure 3).

#### 3.4.3. All-Cause Death

**Figure 4:**
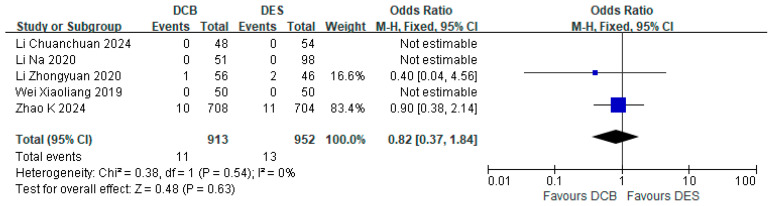
Forest plot of all-cause death [17,18,21,24,26].

Five studies reported the incidence of all-cause death. The DCB group (*n* = 913) experienced 11 all-cause deaths, while the DES group (*n* = 952) had 13 all-cause deaths. The incidence of all-cause death in the DCB group did not significantly exceed that in the DES group (RR = 0.82, 95% CI: [0.37, 1.84]; *p* = 0.63, I^2^ = 0%) (Figure 4).

#### 3.4.4. TVR

**Figure 5:**
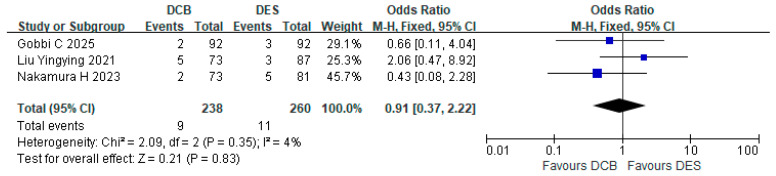
Forest plot of TVR [15,20,22].

Three studies reported the occurrence of TVR. There was a total of 9 TVR events in the DCB group (*n* = 238) and 11 TVR events in the DES group (*n* = 260). The results showed that the incidence of TVR in the DCB group was not higher than that in the DES group (RR = 0.91, 95% CI: [0.37, 2.22], *p* = 0.83, I^2^ = 4%) (Figure 5).

#### 3.4.5. Bleeding Events

**Figure 6:**
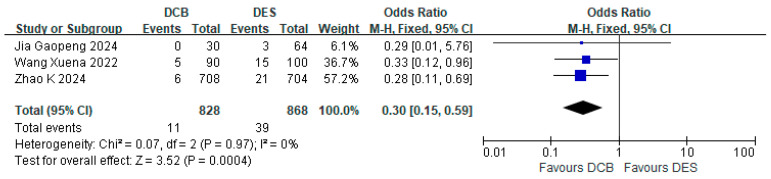
Forest plot of bleeding events [16,23,26].

Three studies reported bleeding events. Of these, two studies adopted a 6-month dual antiplatelet therapy (DAPT) regimen, while one used a 3-month DAPT regimen. There was a total of 11 events in the DCB group (*n* = 828) and 39 TVR events in the DES group (*n* = 868). The results showed that the incidence of bleeding events in the DCB group was lower than that in the DES group (RR = 0.30, 95% CI: [0.15, 0.59], *p* = 0.0004, I^2^ = 0%) (Figure 6).

#### 3.4.6. Restenosis

**Figure 7:**
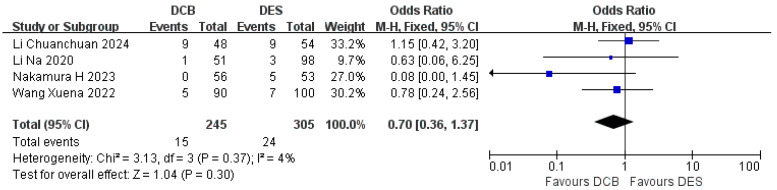
Forest plot of restenosis [17,18,22,23].

Four studies reported the incidence of restenosis. There was a total of 15 restenosis events in the DCB group (*n* = 245) and 24 restenosis events in the DES group (*n* = 305). The incidence of restenosis in the DCB group (*n* = 245) was not higher than that in the DES group (RR = −0.70, 95%CI: [0.36, 1.37], *p* = 0.30, I^2^ = 4%) (Figure 7).

### 3.5. Angiography-Related Secondary Outcomes

#### 3.5.1. Post-Intervention MLD

**Figure 8:**
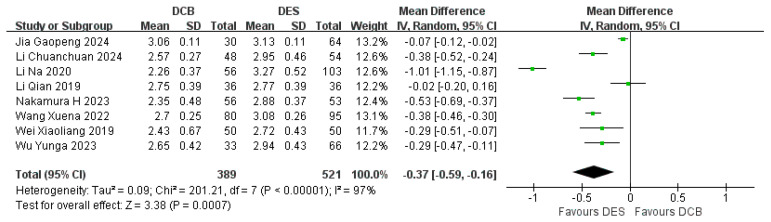
Forest plot of post-intervention MLD [16,17,18,19,22,23,24,25].

Eight studies reported the post-intervention MLD. The results showed that the post-intervention MLD in the DCB group (*n* = 389) was smaller than that in the DES group (*n* = 521) (RR = −0.37, 95% CI: [−0.59, −0.16], *p* = 0.0007, I^2^ = 97%). Because of the high heterogeneity, we used a random effects model to analysis the data (Figure 8).

#### 3.5.2. Follow-Up MLD

**Figure 9:**
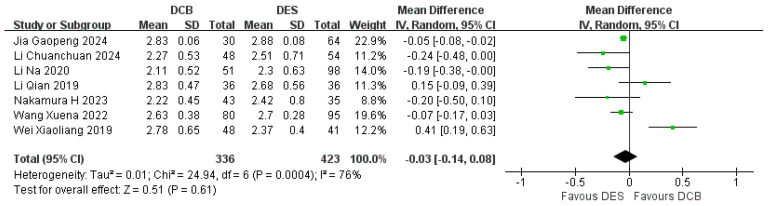
Forest plot of follow-up MLD [16,17,18,19,22,23,24].

Seven studies reported the follow-up MLD. The results showed that the follow-up MLD in the DCB group (*n* = 336) was not less than that in the DES group (*n* = 423). (RR = −0.03, 95% CI: [−0.14, 0.08], *p* = 0.61, I^2^ = 76%, using the random effects model) (Figure 9).

#### 3.5.3. Late Lumen Loss (LLL)

**Figure 10:**
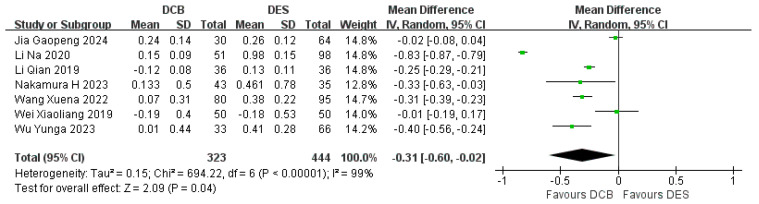
Forest plot of LLL [16,18,19,22,23,24,25].

Eight studies reported the LLL. In the study by Li Chuanchuan et al., LLL did not follow a normal distribution and was not included in the statistics. Combining the results of seven other studies, the DCB group (*n* = 323) had less LLL compared with the DES group (*n* = 444). (RR = −0.31, 95% CI: [−0.60, −0.02], *p* < 0.0001, I^2^ = 99%, using the random effects model) (Figure 10). The results of Li Chuanchuan’s study showed that the LLL of the DCB group (*n* = 48) was 0.19 mm (0.05 mm, 0.30 mm), and the LLL of the DES group (*n* = 54) was 0.25 mm (0.15 mm, 0.39 mm). It is concluded that the LLL of the DCB group was smaller than that of the DES group (Z = −2.171, *p* = 0.03), so not including this research data has no significant impact on the results. It is worth noting that the LLL in studies by Li Qian and Wei Xiaoliang were negative, indicating that the follow-up MLD was larger than the post-intervention MLD.

### 3.6. Subgroup Analyses

#### 3.6.1. Subgroup Analysis of Paclitaxel-Coated Balloons (PCBs)

**Figure 11:**
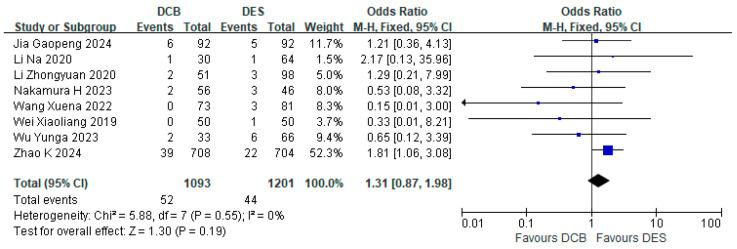
Forest plot of TLR in PCB subgroup [16,18,21,26].

Among the nine studies reporting TLR events, only the Gobbi C trial utilized sirolimus-coated balloons, with the remaining studies employing paclitaxel-coated balloons. The subgroup analysis of paclitaxel-coated balloon studies demonstrated comparable TLR rates: 49 events occurred in the PCB group (*n* = 1093) versus 44 events (*n* = 1021) in the DES group, indicating that TLR incidence with PCB did not exceed that of DES (RR = 1.31, 95% CI: [0.87, 1.98], *p* = 0.19, I^2^ = 0%) (Figure 11).

#### 3.6.2. Subgroup Analysis of Shorter DAPT Duration

**Figure 12:**
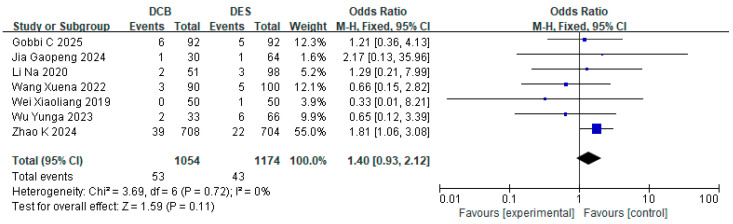
Forest plot of TLR in shorter DAPT subgroup [15,16,18,23,24,25,26].

Among the nine studies reporting TLR outcomes, seven demonstrated shorter DAPT durations in DCB groups versus DES groups. The subgroup analysis of these shorter-DAPT studies revealed comparable TLR incidence: 53 events occurred in DCB cohorts compared with 43 events in DES groups, indicating clinically similar TLR rates (RR = 1.40, 95% CI: [0.93, 2.12], *p* = 0.11, I^2^ = 0%) (Figure 12).

### 3.7. Discussion

#### 3.7.1. Analysis of Results

According to the results, in de novo large coronary lesions, the incidence of clinical events such as TLR, cardiac death, and restenosis, etc., within 1 year after PCI in the DCB group were not inferior to that in the DES treatment group, which indicated that the safety and effectiveness of DCB was comparable to that of DES. Meanwhile, DCB was associated with significantly fewer bleeding events. The post-intervention MLD in the DCB group was smaller than that in the DES group, which may be related to the inability of DCB to provide mechanical support to the coronary vessel wall, resulting in a degree of acute lumen retraction. Although the reported MI events did not distinguish by the time of occurrence, the very low MI incidence rate implied the immediate effectiveness of DCB. It is worth noting that the follow-up MLD in the DCB group was not smaller than that in the DES group, and the LLL in the DCB group was smaller than that in the DES group. In the study by Li Qian and Wei Xiaoliang, the follow-up MLD in the DCB group was even larger than that in the DES group. Regarding this interesting result, whether DCB treatment has long-term effectiveness for de novo large coronary vessel lesions must be discussed.

#### 3.7.2. Analysis of the Restenosis Mechanism of DCB and DES

Nowadays, PCI with DES implantation remains the preferred treatment strategy for CHD. Nevertheless, late-stage (30 days–1 year after intervention) and very-late-stage (>1 year after intervention) ISR and in-stent thrombosis are the main problems affecting the effectiveness of PCI treatment, and these problems increase the incidence of TLR [6]. Therefore, delaying and reducing stent-related TLR is an important issue that needs to be addressed.

Mechanisms of ISR or in-stent thrombosis in late or very late stages include impaired re-endothelialization, late acquired stent malapposition, neoatherosclerosis, and localized hypersensitivity reactions [27]. Despite the renewal of DES, the impaired re-endothelialization remains an unresolved problem. Specifically, physiological functions such as antithrombotic aggregation are limited, which promotes thrombosis and the formation of new atherosclerotic plaque. Moreover, the stent itself can alter local hemodynamics, which promotes platelet activation and thrombus formation [28]. Late acquired stent malapposition is also one of the main mechanisms leading to ISR. Ueki Y et al. found that there was no significant difference in the neointimal hyperplasia between the LLL < 0.5 mm group and the LLL > 0.5 mm group at 6 months to 1 year after sirolimus stent implantation. However, there was a significant difference in the degree of late stent recoil. Also, worse stent adhesion was pronounced in the fibrotic plaque group than in the lipid plaque group [29]. Unlike primary atherosclerosis, the process of neoatherosclerosis can be shortened to months to years after stent implantation. Changes such as lipoprotein infiltration caused by impaired endothelial barrier function, inflammatory cell aggregation caused by drug coating, and fibrin deposition caused by local hemodynamic changes can accelerate the occurrence of atherosclerosis [30,31]. Eosinophils and mast cells can promote the formation of atherosclerotic plaques, reduce plaque stability, shrink coronary vessels, and promote thrombosis, while DES can promote the infiltration of allergic reaction-related cells [32].

DCBs avoid the above pathological processes to some extent. When the DCB expands at the lesion, the anti-proliferative drug transfers to endothelial cells, which does not require the long-term implantation of foreign objects in the body. Thus, DCB avoids long-term hemodynamic changes, avoids the continuous recruitment of inflammatory cells by foreign objects, and avoids the continued influence on endothelial healing. To avoid restenosis, anti-proliferative drugs of DCBs can stay on the vascular wall for a period of time. In an in vitro experiment, although the tissue drug concentration significantly decreased within 24 h after DCB contact with lesions, it tended to stabilize after 72 h [33]. AGENT IDE found that paclitaxel could be detected in a vessel wall within an average of 90 days after DCB intervention [11]. The preceding results implied that in the early and late stages after DCB intervention, anti-proliferative drugs could maintain a certain concentration, which continuously inhibited endometrial hyperplasia, and thus reduced the restenosis rate. In the late and very late stages after DCB intervention, when the concentration of anti-proliferative drugs in the vessel wall drops below the effective concentration, the pro-inflammatory and other damaging effects can be weakened. Comparing this with interventions with DES, the re-endothelial function may be restored earlier and the time of dual antiplatelet therapy may be reduced after DCB treatment. Moreover, different anti-proliferative drug coatings may affect the incidence of late lumen enlargement (LLE). Ahmad WAW et al. compared the use of a sirolimus balloon and a paclitaxel balloon for the treatment of de novo coronary lesions, and the results showed that at 6 months, the angiography results of the two groups were similar, while the proportion of LLE was higher after paclitaxel-coated balloon treatment [34].

Another difference between DCB and DES intervention is that balloon dilation can easily cause dissection. The dissection formed by plain old balloon dilation can lead to early lumen retraction, intimal hyperplasia, and thrombus aggregation, thereby reducing the effective lumen area in the late stage [35]. But DCBs have less early lumen retraction than plain old balloons and could have an enlarged lumen area in the later stages. Cortese B et al. conducted an observational study which included 156 patients with de novo coronary disease treated with DCBs. Among them, 52 patients had post-intervention dissections (with an average RVD of 2.80 mm), of which 48 were A-C type dissections. After an average of 201 days after DCB intervention, follow-up angiography revealed the presence of LLE, and dissections healed in 45 out of 48 patients [36]. The study by Yamamoto et al. also reported that a large-angle dissection (dissection angle > 90°) with stable hemodynamics was associated with LLE [37]. A retrospective study on DCB treating peripheral arterial disease also showed a U-shaped correlation between the risk of restenosis and the angle of dissection, with a cut-off value of 64° indicating the best prognosis [38].

Based on the above mechanism analysis, DCB may exhibit the characteristic of LLE. For CHD, using DCB alone to treat de novo large vessel lesions may show efficacy and safety in the late or very late stages, which may not be inferior to DES.

#### 3.7.3. DCBs with Different Carrier Matrices

The present analysis included only one study utilizing sirolimus-coated balloons (SCBs), and the exclusion of this trial did not substantially alter the primary findings regarding TLR incidence. Furthermore, none of the studies reporting LLL outcomes utilized sirolimus-coated balloons. The EASTBOURNE study is an observational investigation conducted in an SCB-treated population. At 12-month follow-up, TLR occurred in 5.9% of lesions and major adverse cardiovascular events (MACEs) were observed in 9.9% of cases, providing preliminary evidence for the safety and efficacy profile of SCB in treating small vessel disease and ISR [39]. The meta-analysis by Shin et al. compared the efficacy and safety profiles between paclitaxel-coated balloons (PCBs) and SCB. The results demonstrated comparable risks of TLF during 9–12 months follow-up after either PCB or SCB treatment. However, quantitative coronary angiography revealed more favorable MLD outcomes with PCB than SCB, which indicated that PCB might demonstrate superior long-term efficacy compared with SCB [40].

#### 3.7.4. DCB and Fewer Bleeding Events

A DCB-only strategy may shorten DAPT duration, but current guidelines and research lack antiplatelet protocol studies for DCB in large coronary vessels. This meta-analysis concluded that the DCB group had fewer bleeding events than the DES group. Since the peri-PCI antiplatelet regimens were largely similar between the DCB and DES groups across studies, the reduced bleeding in the DCB group may be attributed to its shorter dual antiplatelet therapy (DAPT) duration after PCI. The REC-CAGEFREE II trial demonstrated that among ACS patients treated with DCB only, the stepwise DAPT de-escalation group achieved non-inferior rates of net adverse clinical events (NACEs) and device-oriented composite endpoints (DoCEs) compared with the standard DAPT group, with a significantly lower bleeding risk [41]. In our meta-analysis, the subgroup analysis of shortened DAPT also suggests that treating de novo large coronary vessel lesions with DCB and a shortened DAPT regimen may be safe without compromising TLR outcomes. However, we observed attenuated statistical significance for DCB non-inferiority in this subgroup analysis (*p* = 0.11 vs. primary analysis *p* = 0.27), likely attributable to an elevated ischemic risk associated with shorter DAPT duration. However, longer follow-up and larger sample sizes are needed to confirm this finding.

The optimal antithrombotic regimen for patients requiring anticoagulation after PCI also remains a key research focus. Current guidelines generally recommend the following: (1). prefer dual therapy (OAC + P2Y_12_ inhibitor) over triple therapy (OAC + DAPT); (2). limit the duration and scope of triple therapy; (3). favor NOACs over VKAs. The AUGUSTUS trial (2 × 2 factorial design) demonstrated that in AF patients post-PCI, combining a P2Y_12_ inhibitor with a NOAC (without aspirin or VKA) reduced bleeding risk and cardiovascular hospitalization without increasing ischemic events [42]. An open question is whether DCB-only PCI could further reduce antithrombotic intensity and bleeding risk in such patients. However, none of the included studies in this meta-analysis reported data on atrial fibrillation or anticoagulant use, highlighting a critical gap for future research.

#### 3.7.5. Recent Research

There are also studies not included in the meta-analysis that reflect the efficacy and safety of DCB in treating de novo large vessel lesions.

Some studies included a portion of large vessel samples when comparing the efficacy and safety of DES and DCB. Xue Yu et al. designed a non-inferiority randomized controlled trial, which included 170 patients with coronary RVDs of 2.25 mm to 4 mm. Among them, the population with RVD ≥ 3 mm accounted for 34% and 43% in the DCB and DES groups. The results suggested that there was no significant difference in TLR and other outcomes between the two groups at 12 months. Furthermore, the LLL of the DCB group was negative, indicating the presence of LLE [43].

However, there are also studies that negate the non-inferiority of DCB, such as REC-CAGEFREE I, a randomized non-inferiority study located in China. The primary outcome of this study was DoCE composite outcomes (including cardiac death, target vessel myocardial infarction TV-MI, and CPI-TLR (pathological FFR ≤ 0.8 or stenosis ≥ 70%). Patients were divided into small and non-small vessel groups based on whether the device diameter was less than 3 mm. The non-small vessel group accounted for 50.4% of the DCB group (*n* = 1133) and 46.5% of the DES group (*n* = 1139). During the 24-month follow-up, the incidence of DoCE in the DCB group was higher than that in the DES group (6.4% vs. 3.4%, *p* = 0.0008), and the incidence of CPI-TLR in the DCB group was also higher than that in the DES group (3.1% vs. 1.2%, *p* = 0.0021). In non-small vessel lesions, the 2-year cumulative DoCE incidence rate was 7.5% in the DCB group and 2.5% in the DES group, indicating that DCB did not demonstrate its non-inferiority compared with DES in non-small vessel lesions [44].

There are also studies comparing the efficacy and safety of DCB in the treatment of large and small vessel lesions, and it was found that DCB has similar efficacy and safety in treating both large and small vessel lesions. Rosenburg et al. divided de novo coronary lesions into large and small vessel groups with a 2.75 mm boundary, and used DCB only as treatment. The results showed that there was no statistically significant difference in clinical events such as TLR, MACE, and restenosis rate after the 9-month follow-up, and only one case of TLR occurred in the large vessel group [45].

#### 3.7.6. Limitations and Prospects

The studies and RCTs included in this meta-analysis are insufficient, and the sample size of clinically important statistical data such as TLR and restenosis rate is small and therefore cannot fully evaluate the efficacy of DCBs. Secondly, the study samples are mainly from the Asian population; therefore, the efficacy of DCBs in other areas cannot be confirmed yet. Moreover, the follow-up time of included studies is around 6 months to 1 year, which cannot confirm that the efficacy of DCBs in treating de novo large coronary lesions is not inferior to DESs in the very late stage.

Based on the analysis of this meta-analysis and existing research, for de novo large coronary artery disease, DCBs have the advantage of having no permanent implant, which reduce long-term damage to endothelial functions. In addition, proliferative drugs can stay in the vessel wall for a period of time to inhibit intimal hyperplasia and prevent restenosis. The above two points constitute the advantages of DCBs in treating de novo coronary artery disease. At present, DCB is not inferior to DES in the treatment of ISR, small vessel disease, and bifurcation disease, and has a shorter DAPT time. Therefore, for patients who have contraindications to stent implantation or need to shorten the DAPT time, DCB has certain advantages compared with DES. Therefore, it is necessary to further evaluate the long-term efficacy and safety of DCB treatment in de novo large coronary lesions, and DCB is expected to become the preferred treatment device for CHD. However, due to insufficient research on the pathological and pharmacokinetic mechanisms of DCB treatment, it is not yet possible to predict the long-term effects of DCB on de novo large coronary lesions. Although this article analyzes LLE and its possible mechanisms with DCBs, whether this phenomenon can lead to a decrease in the incidence of clinical events such as very-late-stage TLR and MACE needs to be further investigated. This article also mentions that dissection formation may be one of the mechanisms underlying LLE, and how the extent, angle, and degree of dissections may affect LLE needs further study. In addition, RVD itself may have an impact on the occurrence of LLE. In addition, the duration of the effective concentration of anti-proliferative drugs in the vessel wall is unclear. How to balance the inhibition of endothelial growth and the promotion of endothelial function restoration deserves further study.

## 4. Conclusions

In summary, this meta-analysis confirms that in the early and late stages after PCI, DCB is not inferior in efficacy and safety to DES for de novo large coronary lesions with RVD > 2.75 mm.

## Figures and Tables

**Table 1:** Baseline characteristics of included studies.

**Study**		Gobbi C, 2025 [15]	Jia Gaopeng, 2024 [16]	Li Cuanchuan, 2024 [17]	Li Na, 2020 [18]	Li Qian, 2019 [19]	Liu Yingying, 2021 [20]	Li Zhongyuan, 2020 [21]	Nakamura H 2023 [22]	Wang Xuena, 2022 [23]	Wei Xiaoliang, 2019 [24]	Wu Yunga, 2023 [25]	Zhao K, 2024 [26]
**Research type**		Retrospective cohort study	Retrospective cohort study	Retrospective cohort study	Retrospective cohort study	Retrospective cohort study	Retrospective cohort study	Retrospective cohort study	Retrospective cohort study	Retrospective cohort study	RCT	Retrospective cohort study	Retrospective cohort study
**Included population**		Patients with de novo lesions in large coronary vessels	CHD, age > 60	CHD, de novo long lesion of large vessels	At least one coronary vessel has stenosis >50%	Age ≥ 65 years, ≤75 years; CHD patients diagnosed with stable and unstable angina pectoris	CHD with diabetes	CHD, age ≥ 60 years	CHD population undergoing PCI	CHD	CHD population with chest pain	CHD	Patients with de novo lesions in large coronary vessels
**RVD**		>2.75 mm	>2.75 mm	≥2.75 mm, ≤5 mm	≥2.8 mm	≥2.8 mm	≥3.0 mm	≥2.8 mm	≥2.75 mm	≥2.8 mm	≥2.8 mm	≥2.8 mm	≥3.0 mm
**DCB group** **(*n* =)**		92	30	48	56	36	87	56	73	90	50	33	708
**DES group** **(*n* =)**		92	64	54	103	36	73	46	81	100	50	66	704
**Follow-up time**		DCB group 19.5 ± 12 months, DES group 20.1 ± 13.1 months	1 year	1 year	6 months	6 months	1 year	1 year	DCB group 8–12 months, DES group 9–14 months	1 year	14.7 ± 4.2 months	1 year	2 years
**Antiplatelet therapy**	**DCB**	DAPT for 3 months	DAPT for 6 months	Not mentioned	DAPT for 6 months	A + T for 3 months, followed by aspirin monotherapy	DAPT for 3 months	DAPT for 1 year	Not randomized	DAPT for 3 months	Not mentioned	DAPT for 6 months	DAPT for 6 months
	**DES**	DAPT for 6 months	DAPT for 1 year		DAPT for 1 year	Aspirin+ Ticagrelor for 1 year, followed by aspirin monotherapy	DAPT for 1 year	DAPT for 1 year		DAPT for 1 year		DAPT for 1 year	DAPT for 1 year
**Equipment information**	**DCB**	SCB (MagicTouch)	PCB	Not mentioned	PCB (Qingzhou)	Not mentioned	Not mentioned	PCB	PCB (Sequent Please)	PCB (Sequent Please, Swide)	PCB (Sequent Please)	PCB	PCB (Sequent Please, bingo, Vesselin, RESTORE DEB, Swide)
	**DES**	Second-generation DES	Second-generation DES		Second-generation DES			Everolimus DES	15 patients used the second-generation DES, 66 patients used the third-generation DES	Sirolimus DES	Not mentioned	Not mentioned	Second-generation DES
**Outcome**		TLR, TVR, MI, cardiac death, TLF	MACE (recurrence angina, MI, cardiac death, TLR, bleeding events), MLD, AG, RDS, LLL, DS	MACE (restenosis, angina, MI, all-cause mortality), MLD, LLL	MACE (nonfatal MI, all-cause mortality, TLR), restenosis, MLD, LLL	MACE (MI, HF, cardiogenic shock, cardiac death), MLD, LLL	MACE (nonfatal MI, ischemic stroke, cardiac death, stent thrombosis, TVR)	MACE (all-cause mortality, LLL, MI, TLR), MLD, restenosis	TLF (cardiac death, nonfatal MI, TVR, TLR), all-cause mortality, restenosis, LLE, MLD, LLL, AG	MACE (MI, cardiac death, TLR), bleeding events, restenosis, AG, MLD, LLL, PB, MLA	MACE (all-cause mortality, nonfatal MI, TLR), MLD, LLL	MACE (MI, fatal arrhythmia, cardiac death, HF, TLR), LLL	MACE (TLR, MI, cardiac death), all-cause death, bleeding events

MI, myocardial infarction; AG, acute luminal benefit; RDS, residual diameter stenosis rate, RDS = (1-MLD/RVD) × 100%; DS, diameter stenosis rate, DS = MLD/RVD × 100%; LLE, negative late-stage lumen loss rate; PB, plaque burden; MLA, the minimum luminal area of the target lesion; HF, heart failure; DAPT, Dual Antiplatelet Therapy; PCB, paclitaxel-coated balloon; SCB, sirolimus-coated balloon.

**Table 2:** Analysis of the risk of bias for each included study according to NOS.

Research	Selection				Comparability	Outcome			Quality Score
	Representatives of the Exposed Cohort	Selection of the Non-Exposed Cohort	Ascertainment of Exposure	Demonstration That Outcome of Interest Was Not Present at Start of Study		Assessment of Outcome	Was Follow-Up Long Enough for Outcomes to Occur	Adequacy of Follow Up of Cohorts	
Gobbi C, 2025 [15]	**★**	★	★	★	★★	★	☆	★	8
Jia Gaopeng, 2024 [16]	★	★	★	★	★★	★	★	★	9
Li Cuanchuan, 2024 [17]	★	★	★	★	★★	☆	★	★	8
Li Na, 2020 [18]	★	★	★	★	★★	★	☆	☆	7
Li Qian, 2019 [19]	★	★	★	★	★★	☆	☆	☆	6
Liu Yingying, 2021 [20]	★	★	★	★	★★	☆	★	☆	7
Li Zhongyuan, 2020 [21]	★	★	★	☆	★★	★	★	★	8
Nakamura H, 2023 [22]	★	☆	★	★	★★	★	★	☆	7
Wang Xuena, 2022 [23]	★	★	★	★	★★	★	★	☆	8
Wei Xiaoliang, 2019 [24]	★	★	★	★	★★	★	★	☆	8
Wu Yunga, 2023 [25]	★	★	★	★	★	☆	★	★	7
Zhao K, 2024 [26]	★	★	★	★	★★	★	★	★	9

‘★’ represents that the study scored 1 point in that aspect; ‘★★’ represents that the study scored 2 points in that aspect; while ‘☆’ represents that the study scored 0 points in that aspect.

## Data Availability

Not applicable.

## References

[1] Ulbricht T.L.V., Southgate D.A.T. (1991). Coronary heart disease: Seven dietary factors. Lancet.

[2] Malakar A.K., Choudhury D., Halder B., Paul P., Uddin A., Chakraborty S. (2019). A review on coronary artery disease, its risk factors, and therapeutics. J. Cell. Physiol..

[3] Grüntzig A.R., Senning Å., Siegenthaler W.E. (1979). Nonoperative dilatation of coronary-artery stenosis. N. Engl. J. Med..

[4] Babapulle M.N., Eisenberg M.J. (2002). Coated stents for the prevention of restenosis: Part II. Circulation.

[5] Virmani R., Liistro F., Stankovic G., Di Mario C., Montorfano M., Farb A., Kolodgie F.D., Colombo A. (2002). Mechanism of late in-stent restenosis after implantation of a paclitaxel derivate–eluting polymer stent system in humans. Circulation.

[6] Giustino G., Colombo A., Camaj A., Yasumura K., Mehran R., Stone G.W., Kini A., Sharma S.K. (2022). Coronary in-stent restenosis. JACC.

[7] Ge J.-B., Chen Y.-D. (2024). Chinese expert consensus on the clinical application of drug-coated balloon (2nd Edition). J. Geriatr. Cardiol..

[8] Scheller B., Speck U., Abramjuk C., Bernhardt U., BöhM M., Nickenig G. (2004). Paclitaxel balloon coating, a novel method for prevention and therapy of restenosis. Circulation.

[9] Speck U., Scheller B., Abramjuk C., Breitwieser C., Dobberstein J., Boehm M., Hamm B. (2006). Neointima inhibition: Comparison of effectiveness of non–stent-based local drug delivery and a drug-eluting stent in porcine coronary arteries. Radiology.

[10] Scheller B., Hehrlein C., Bocksch W., Rutsch W., Haghi D., Dietz U., Böhm M., Speck U. (2008). Two year follow-up after treatment of coronary in-stent restenosis with a paclitaxel-coated balloon catheter. Clin. Res. Cardiol..

[11] Yeh R.W., Shlofmitz R., Moses J., Bachinsky W., Dohad S., Rudick S., Stoler R., Jefferson B.K., Nicholson W., Altman J. (2024). Paclitaxel-coated balloon vs uncoated balloon for coronary in-stent restenosis. JAMA.

[12] Byrne R.A., Joner M., Alfonso F., Kastrati A. (2013). Drug-coated balloon therapy in coronary and peripheral artery disease. Nat. Rev. Cardiol..

[13] Latib A., Colombo A., Castriota F., Micari A., Cremonesi A., De Felice F., Marchese A., Tespili M., Presbitero P., Sgueglia G.A. (2012). A randomized multicenter study comparing a paclitaxel drug-eluting balloon with a paclitaxel-eluting stent in small coronary vessels. JACC.

[14] Jeger R.V., Farah A., Ohlow M.-A., Mangner N., Möbius-Winkler S., Weilenmann D., Wöhrle J., Stachel G., Markovic S., Leibundgut G. (2020). Long-term efficacy and safety of drug-coated balloons versus drug-eluting stents for small coronary artery disease (BASKET-SMALL 2): 3-year follow-up of a randomised, non-inferiority trial. Lancet.

[15] Gobbi C., Giangiacomi F., Pasero G., Faggiano A., Barbieri L., Tumminello G., Colombo F., Ruscica M., Ardizzone V., Genta E. (2025). Efficacy and safety of sirolimus-coated balloon angioplasty in de novo lesions in large coronary vessels: A propensity score-matched study. Catheter. Cardiovasc. Interv..

[16] Jia G., Qu Z., Li G., Huangfu W., Zhao Z., Yan S., Chen Q., Zhang Y. (2024). Efficacy and safety of drug coated balloon in the treatment of coronary artery lesions in situ in elderly patients: A cohort study. Chin. Gen. Pract..

[17] Li C.-C., Lin W.-Y., Lin C.-H., Chen C.-Y., Cheng C.-I., Chen Y.-H., Lee W.-L. (2024). The safety and efficacy of drug coated balloon in the treatment of uncomplicated large coronary artery in situ lesions. J. Appl. Electrocardiol..

[18] Li N. (2020). Clinical Study of Drug Coated Balloon in the Treatment of Primary Lesions of Coronary Artery In Situ. M.A. Thesis.

[19] Li Q., Chang S., Zhang Y., Wang X., Zhang Y., Chen Q. (2019). A comparative study of the efficacy of drug-eluting stents and drug-eluting balloons in the treatment of primary lesions in situ of large coronary arteries. Chin. J. Geriatr. Heart Brain Vessel Dis..

[20] Liu Y. (2021). Efficacy of Drug Coated Balloon in Diabetic Coronary Artery In Situ Macroangiopathy. M.A. Thesis.

[21] Li Z. (2020). Application of drug coated balloon in interventional treatment of primary lesions in situ of coronary artery in the elderly. Chin. Convalescent Med..

[22] Nakamura H., Ishikawa T., Mizutani Y., Yamada K., Ukaji T., Kondo Y., Shimura M., Aoki H., Hisauchi I., Itabashi Y. (2023). Clinical and angiographic outcomes of elective paclitaxel-coated balloon angioplasty in comparison with drug-eluting stents for de novo coronary lesions in large vessels. Int. Heart J..

[23] Wang X. (2022). Clinical Efficacy of Drug Coated Balloon in the Treatment of Coronary Artery Lesions In Situ. M.A. Thesis.

[24] Wei X., Wang X., Zhao T., Lin X., Chen X. (2019). Study on the efficacy and safety of paclitaxel drug coated balloon in the treatment of primary coronary artery disease. Chin. J. Arterioscler..

[25] Wu Y. (2023). Application Effect of Drug Balloon Only in the Treatment of Primary Coronary Artery Large Vessel Lesions. M.A. Thesis.

[26] Zhao K., Guo Q., Zhao Z., Tang H., You R., Peng L., Rao L., Li M. (2024). Clinical value of drug-coated balloon versus second-generation drug-eluting stent for de novo lesions in large coronary arteries: Insights from the real world. BMC Cardiovasc. Disord..

[27] Torrado J., Buckley L., Durán A., Trujillo P., Toldo S., Raleigh J.V., Abbate A., Biondi-Zoccai G., Guzmán L.A. (2018). Restenosis, Stent Thrombosis, and Bleeding Complications. JACC.

[28] Otsuka F., Finn A.V., Yazdani S.K., Nakano M., Kolodgie F.D., Virmani R. (2012). The importance of the endothelium in atherothrombosis and coronary stenting. Nat. Rev. Cardiol..

[29] Ueki Y., Räber L., Otsuka T., Rai H., Losdat S., Windecker S., Garcia-Garcia H.M., Landmesser U., Koolen J., Byrne R. (2020). Mechanism of drug-eluting absorbable metal scaffold restenosis. Circ. Cardiovasc. Interv..

[30] Otsuka F., Byrne R.A., Yahagi K., Mori H., Ladich E., Fowler D.R., Kutys R., Xhepa E., Kastrati A., Virmani R. (2015). Neoatherosclerosis: Overview of histopathologic findings and implications for intravascular imaging assessment. Eur. Heart J..

[31] Torii S., Jinnouchi H., Sakamoto A., Kutyna M., Cornelissen A., Kuntz S., Guo L., Mori H., Harari E., Paek K.H. (2019). Drug-eluting coronary stents: Insights from preclinical and pathology studies. Nat. Rev. Cardiol..

[32] Niccoli G., Montone R.A., Sabato V., Crea F. (2018). Role of allergic inflammatory cells in coronary artery disease. Circulation.

[33] Gray W.A., Granada J.F. (2010). Drug-Coated Balloons for the Prevention of Vascular Restenosis. Circulation.

[34] Ahmad W.A.W., Nuruddin A.A., Kader M.A.S.A., Ong T.K., Liew H.B., Ali R.M., Zuhdi A.S.M., Ismail M.D., Yusof A.K., Schwenke C. (2022). Treatment of Coronary De Novo Lesions by a Sirolimus- or Paclitaxel-Coated Balloon. JACC Cardiovasc. Interv..

[35] Jinnouchi H., Sakakura K., Yamamoto K., Taniguchi Y., Fujita H. (2023). A unique mechanism of restenosis after drug-coated balloon in peripheral artery: Insight from optical frequency domain imaging. Cardiovasc. Revascularization Med..

[36] Cortese B., Orrego P.S., Agostoni P., Buccheri D., Piraino D., Andolina G., Seregni R.G. (2015). Effect of drug-coated balloons in native coronary artery disease left with a dissection. JACC Cardiovasc. Interv..

[37] Yamamoto T., Sawada T., Uzu K., Takaya T., Kawai H., Yasaka Y. (2020). Possible mechanism of late lumen enlargement after treatment for de novo coronary lesions with drug-coated balloon. Int. J. Cardiol..

[38] Kozuki A., Takahara M., Shimizu M., Kijima Y., Nagoshi R., Fujiwara R., Shibata H., Suzuki A., Soga F., Miyata T. (2021). Outcomes of Dissection Angles as Predictor of Restenosis after Drug-Coated Balloon Treatment. J. Atheroscler. Thromb..

[39] Cortese B., Testa L., Heang T.M., Ielasi A., Bossi I., Latini R.A., Lee C.Y., Perez I.S., Milazzo D., Caiazzo G. (2023). Sirolimus-coated balloon in an all-comer population of coronary artery disease patients. JACC Cardiovasc. Interv..

[40] Shin D., Singh M., Shlofmitz E., Scheller B., Latib A., Kandzari D.E., Zaman A., Mylotte D., Dakroub A., Malik S. (2024). Paclitaxel-coated versus sirolimus-coated balloon angioplasty for coronary artery disease: A systematic review and meta-analysis. Catheter. Cardiovasc. Interv..

[41] Gao C., Zhu B., Ouyang F., Wen S., Xu Y., Jia W., Yang P., He Y., Zhong Y., Zhou Y. (2025). Stepwise dual antiplatelet therapy de-escalation in patients after drug coated balloon angioplasty (REC-CAGEFREE II): Multicentre, randomised, open label, assessor blind, non-inferiority trial. BMJ.

[42] Berwanger O., Wojdyla D.M., Fanaroff A.C., Budaj A., Granger C.B., Mehran R., Aronson R., Windecker S., Goodman S.G., Alexander J.H. (2024). Antithrombotic Strategies in Atrial Fibrillation After ACS and/or PCI. J. Am. Coll. Cardiol..

[43] Yu X., Wang X., Ji F., Zhang W., Yang C., Xu F., Wang F. (2021). A Non-inferiority, Randomized Clinical Trial Comparing Paclitaxel-Coated Balloon Versus New-Generation Drug-Eluting Stents on Angiographic Outcomes for Coronary De Novo Lesions. Cardiovasc. Drugs Ther..

[44] Gao C., He X., Ouyang F., Zhang Z., Shen G., Wu M., Yang P., Ma L., Yang F., Ji Z. (2024). Drug-coated balloon angioplasty with rescue stenting versus intended stenting for the treatment of patients with de novo coronary artery lesions (REC-CAGEFREE I): An open-label, randomised, non-inferiority trial. Lancet.

[45] Rosenberg M., Waliszewski M., Krackhardt F., Chin K., Ahmad W.A.W., Caramanno G., Milazzo D., Nuruddin A.A., Liew H.B., Maskon O. (2019). Drug coated balloon-only strategy in de novo lesions of large coronary vessels. J. Interv. Cardiol..

